# Robot‐Assisted Versus Open Surgery for the Resection of Large Thymomas: A Retrospective Cohort Study

**DOI:** 10.1002/jso.28113

**Published:** 2025-03-28

**Authors:** Benedikt Niedermaier, Raffaella Griffo, Florian Eichhorn, Laura V. Klotz, Heidrun Grosch, Thomas Muley, Hauke Winter, Martin E. Eichhorn

**Affiliations:** ^1^ Department of Thoracic Surgery Thoraxklinik at the University of Heidelberg Heidelberg Germany; ^2^ Translational Lung Research Center Heidelberg (TLRC‐H) Member of the German Center for Lung Research Heidelberg Germany; ^3^ Translational Research Unit Thoraxklinik at University Hospital Heidelberg Heidelberg Germany; ^4^ Department of Oncology Thoraxklinik at the University of Heidelberg Heidelberg Germany

**Keywords:** mediastinum, robotic surgery, survival, thymic tumors, thymoma

## Abstract

**Background:**

Robot‐assisted thoracic surgery (RATS) is increasingly becoming the preferred surgical method for the resection of thymomas. We initiated the current study to evaluate perioperative outcomes and early recurrence associated with the surgical approach.

**Methods:**

In this retrospective cohort study, 35 patients were included in the RATS group and compared with 29 patients who underwent open surgery between 2010 and 2022 for histologically confirmed large thymoma > 50 mm in TNM Stages I, II, and IIIa.

**Results:**

Histologic subtypes and pathologic stages were similar in both groups. The median duration of chest drainage and median length of stay was significantly shorter in the RATS group (1 vs. 4 days, *p* < 0.0001, and 4 vs. 10 days, *p* < 0.0001, respectively). Postoperative complications occurred more frequently in the open surgery group (cumulative incidence of 8.6% vs. 37.9%, *p* = 0.0048). The median follow‐up time was 28 months in the RATS group and 69 months in the open surgery group. Five‐year recurrence‐free survival was 96.9% without significant differences between the two groups.

**Conclusion:**

There is no evidence of increased early recurrence associated with robotic surgery. The length of hospital stay, the duration of thoracic drainage, and the significantly lower complications favor the robot‐assisted approach.

## Summary

- Robot‐assisted resection of large thymomas > 5 cm is not associated with increased early recurrence compared to open surgery.
- Shorter hospital stays and shorter chest tube duration as well as fewer complications were observed after robot‐assisted resection.
- The perioperative advantages with similar oncologic outcomes favor the robotic approach.

**Abbreviations**
IASLC: The International Association for the Study of Lung Cancer
IQR: interquartile range
ITMIG: International Thymic Malignancy Interest Group
RATS: robot‐assisted thoracic surgery

## 1 Introduction

Thymoma is the most common malignant tumor of the anterior mediastinum with an estimated annual incidence of 2−3 cases per million [1, 2]. Different histological subtypes are distinguished: type A, AB thymoma, and type B thymoma, subdivided into B1, B2, and B3 thymomas [3, 4]. Surgery is the recommended treatment for localized disease, and the prognosis after complete resection is good [5]. Traditionally, open surgery by sternotomy or thoracotomy has been the preferred surgical approach, ensuring complete resection with adequate safety margins [5, 6]. In the last two decades, minimally invasive surgical techniques, including video‐assisted and robotic surgery, have rapidly developed as alternatives to traditional open surgery [7, 8, 9]. Robot‐assisted thoracic surgery (RATS) offers advantages over open surgery such as better cosmetic results, faster recovery of lung function, less surgical trauma, shorter hospital stays, and fewer complications [8, 10, 11, 12]. However, the limits of maximum resectable tumor size and oncological results are still controversial. From a technical perspective, large tumors can restrict the surgeon's field of vision during minimally invasive procedures and obstruct access to vital surrounding structures, such as the major mediastinal vessels. In addition, critics have raised concerns that capsular rupture and tumor manipulation may affect the risk of local recurrence [13]. As a result, the adoption of RATS for thymoma resection has been debated controversially as the oncologic and procedure‐related complications have not been clearly identified. In view of these developments, there is a need to evaluate survival and recurrence risk after thymoma resection using RATS compared to open surgery.

## 2 Patients and Methods

### 2.1 Patients

This retrospective study was conducted on the basis of a prospectively compiled database comprising all patients who have undergone robot‐assisted surgery for a thymic tumor since 2018. The study was approved by the ethics committee of Heidelberg University. Written informed consent was obtained from all patients before surgery. All patients who underwent primary robot‐assisted surgery for thymoma between 2018 and 2022 were identified. Patients with histologically confirmed thymoma in TNM Stages I, II, and IIIa with the largest diameter of 50 mm or more were included in this study. Pathologic staging was reported according to the Masaoka−Koga classification and the 8th edition of the TNM classification as proposed by the IASLC/ITMIG Thymic Epithelial Tumors Staging Project [14]. Records from before the introduction of this classification were reclassified accordingly. Exclusion criteria were thymic carcinoma histology and other histological types of anterior mediastinal tumors as well as prior neoadjuvant chemotherapy.

To compare perioperative and oncologic outcomes of RATS to the traditional open approach, patients with thymoma undergoing primary resection by thoracotomy or sternotomy from 2010 to 2022 were included in the control group. Since all patients in the robotic group had histologically confirmed negative margins, any patients with R1 resection were excluded from the control group for better comparability. Medical histories, surgical records, radiological data, and pathological results were reviewed. Clinical information such as age, gender, height, weight, and symptoms were recorded, as well as histological features according to the WHO classification. Surgical records were reviewed to assess the site of access, operating times, and reasons for conversion.

### 2.2 Preoperative Management

Patients with suspected thymomas were discussed by a multidisciplinary board including a pneumologist, a thoracic oncologist, a thoracic surgeon, and a radiologist. Indication for surgery was made when resection appeared technically and clinically feasible for suspected TNM Stages I, II, and IIIa. After RATS became available in 2018, the main criteria for choosing between open surgery and RATS were suspected invasion into surrounding structures and size. While resection of the pericardium or lung can be safely handled in RATS, we preferred the open approach for suspected invasion of the superior vena cava and pulmonary artery trunk. Of note, as expertise with RATS grew, continuously larger and more advanced tumors were considered feasible for RATS, and even tumors > 100 mm were eventually scheduled for RATS.

Before surgery, myasthenia gravis (MG) symptoms were routinely evaluated and serological diagnostics performed if necessary. For patients with MG, medical management was implemented if not already established, and surgery only performed after achieving clinically stable disease. Patients with pure red cell aplasia (PRCA) received blood transfusions if necessary.

### 2.3 Surgical Technique

All operations in the RATS group were performed using the DaVinci X system (Intuitive Surgical, Sunnyvale, CA, USA) with a dual console. Surgery was performed under single‐lung ventilation with a double‐lumen endotracheal tube and intrathoracic CO_2_ insufflation at a pressure of 8−12 mmHg. Mediastinal tumors were removed using robotic‐assisted techniques from either the right or left side, depending on their location. The authors preferred a left‐sided approach for left‐sided or central tumors and a right‐sided approach for predominantly right‐sided tumors. The procedure required precise positioning of the patient and placement of the trocars to maximize robotic arm mobility and avoid collisions. Typically, a three‐arm technique was used in combination with a 12 mm auxiliary port in the 8th intercostal space and with 3 × 8 mm trocars placed laterally to introduce the video optics and robotic instruments. At the beginning of the operation, the pleura was incised pleurally along the sternum and the phrenic nerve on the left and right. Smaller vessels were coagulated bipolar, and larger vessels were closed robotically with self‐locking clips. In the case of pericardial tumor infiltration, the pericardium was partially resected. The tumor was removed through the caudal assist port incision, which was enlarged for larger tumors. For extensive resections involving the lung, phrenic nerve, or pericardial resection, a fourth arm was inserted for better exposure. The tumor was removed en bloc with the surrounding tissue, ensuring R0 resection. If larger parts of the pericardium had to be removed and there was a risk of heart dislocation, it was replaced by bovine pericardium.

In the control group with open surgery, a sternotomy or an anterolateral thoracotomy was chosen as the surgical approach to the mediastinum, depending on the size and location of the tumor. Resection of the thymoma was performed according to a standard procedure. The thymoma was mobilized, taking into account the surrounding pericardial fatty tissue and the surrounding tissue and adjacent structures such as the phrenic nerve, the pericardium, the large vessels, and the lungs. If the vena cava was infiltrated, it was replaced by a tubular prosthesis.

Patients were routinely extubated in the OR. Depending on the estimated perioperative risk, considering the invasiveness of approach, age and comorbidities, patients were either transported to the recovery room and surveilled until fully awake, or transported to the intensive care unit for 1 day of intended postoperative surveillance.

### 2.4 Follow‐Up

Postoperative staging was performed at 6‐month intervals for the first 5 years after surgery, followed by annual check‐ups for a further 5 years. During the follow‐up visit, a medical history was taken, a physical examination was performed, and a contrast‐enhanced CT scan and a pulmonary function test were carried out. The patients and their attending physicians were contacted in the case of patients whose follow‐up care took place outside the hospital. In case of ambiguous external assessment, CT images were subsequently sent to our center to be discussed with the multidisciplinary board.

### 2.5 Statistical Analysis

Visual representation and statistical analysis were conducted using Prism (Version 8.2.1, Graphpad Software Inc, Boston, USA). Patients who underwent conversion from a robotic to an open surgical procedure were analyzed in the RATS group based on intention to treat. Demographic, clinical, and pathological characteristics were presented in a descriptive analysis, with continuous variables expressed as median with interquartile range and categorical variables as number and percentage. The RATS and open surgery group were compared using the Chi‐square test for categorical variables and the Mann−Whitney test for continuous variables. *p* values below 0.05 were considered significant. Overall survival and recurrence‐free survival was estimated using the Kaplan−Meier method with the log‐rank (Mantel−Cox) test.

## 3 Results

### 3.1 Demographics and Pathology

**Figure 1:**
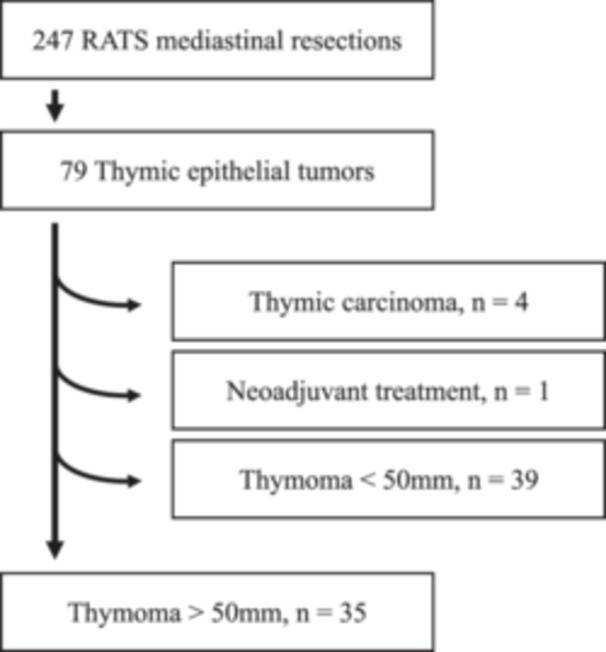
Flowchart for the selection of patients included in the study.

In this retrospective cohort study, 35 consecutive patients were included in the RATS group according to selection criteria (Figure 1). Twenty‐nine consecutive patients who underwent open resection were included in the open surgery control group. Detailed demographic and clinical data is given in Table 1. The median age at the time of surgery was 60 years, with no significant differences between the two groups. Altogether, 33 male and 31 female patients were included, but balance between the RATS and open surgery groups significantly differed with more female patients in the RATS group and more male patients in the open surgery group (*p* = 0.0112). Twenty patients (57.1%) in the RATS group were never‐smokers compared to 14 patients (48.3%) in the open surgery group; overall, no differences were observed in smoking history. Thymoma‐associated immune pathology was observed in 18 patients (29.1%). The observed incidence was balanced between the two groups without statistically significant differences. Nine patients (14.1%) were diagnosed with MG, seven patients (10.9%) with immunodeficiency, and two patients (3.1%) displayed PRCA. Aside from symptoms related to the respective autoimmune disease, 62.5% of the patients presented with no specific symptoms, 14.1% of the patients complained of dyspnea, 6.3% of cough, and 1.6% of chest pain.

**Table 1:** Demographic and clinical data.

	Total	RATS	Open surgery	*p* value
Patients, *n*	64	35	29	
Age, years	60 (52−67)	61 (56−72)	59 (51−67)	0.2024
Gender				**0.0112**
Male	33 (51.6%)	13 (37%)	20 (69%)	
Female	31 (48.4%)	22 (63%)	9 (31%)	
BMI	27 (23−30)	25 (23−28)	27 (24−33)	0.0624
Smoking history				0.7785
Never	34 (53.1%)	20 (57.1%)	14 (48.3%)	
Former	24 (37.5%)	12 (34.3%)	12 (41.4%)	
Current	6 (9.4%)	3 (8.6%)	3 (10.3%)	
Autoimmune disease				0.5231
None	46 (71.9%)	27 (77.1%)	19 (65.5%)	
Myasthenia gravis	9 (14.1%)	5 (14.3%)	4 (13.8%)	
Immunodeficiency	7 (10.9%)	2 (5.7%)	5 (17.2%)	
PRCA	2 (3.1%)	1 (2.9%)	1 (3.4%)	
Symptoms^a^				0.2878
None	40 (62.5%)	26 (74.3%)	14 (48.3%)	
Dyspnea	9 (14.1%)	3 (8.6%)	6 (20.7%)	
Cough	4 (6.3%)	2 (5.7%)	2 (6.9%)	
Pain	1 (1.6%)	1 (2.9%)	0 (0%)	
Related to autoimmune disease	18 (28.1%)	8 (22.9%)	10 (34.5%)	

*Note:* Demographic and clinical data. Values are given as median (interquartile range) or *n* (%). *p* values show the result of the Chi‐square test for categorical variables and the Mann−Whitney test for continuous variables comparing RATS and open surgery. Bold values indicate statistically significant.

Abbreviations: BMI, body mass index; PRCA, pure red cell aplasia.

^a^ Categories are not mutually exclusive.

Tumors were at least 50 mm in the largest diameter, measured during pathological examination. Median tumor size was 68 mm in the RATS group and 99 mm in the open surgery group (*p *= 0.0012). Detailed information on pathology is given in Table 2. The composition of the groups was similar with regard to the histological subtypes defined by the WHO. There were no significant differences in tumor stage according to the Masaoka−Koga staging system and the IASLC/ITMIG system (TNM 8th edition).

**Table 2:** Pathologic results.

	Total	RATS	Open surgery	*p* value
Tumor size, mm	81 (62−103)	68 (57−90)	99 (74.5−132)	**0.0012**
WHO classification				0.1928
A	14 (16.5%)	11 (31.4%)	3 (10.3%)	
AB	27 (42.2%)	11 (31.4%)	16 (55.2%)	
B1	7 (10.9%)	5 (14.3%)	2 (6.9%)	
B2	9 (14.1%)	4 (11.4%)	5 (17.2%)	
B3	1 (1.6%)	1 (2.9%)	0	
B mixed	6 (9.4%)	3 (8.6%)	3 (10.3%)	
Masaoka−Koga stage				0.2265
I	31 (48.4%)	17 (48.6%)	14 (48.3%)	
IIa	19 (29.7%)	13 (37.1%)	6 (20.7%)	
IIb	8 (12.5%)	2 (5.7%)	6 (20.7%)	
III	6 (9.4%)	3 (8.6%)	3 (10.3%)	
TNM stage				0.5301
I	58 (90.6%)	32 (91.4%)	26 (89.7%)	
II	1 (1.6%)	1 (2.9%)	0	
IIIa	5 (7.8%)	2 (5.7%)	3 (10.3%)	

*Note:* Pathologic results. Values are given as median (interquartile range) or *n* (%). *p* values show the result of the Chi‐square test for categorical variables and the Mann−Whitney test for continuous variables. Bold values indicate statistically significant.

### 3.2 Operation and Perioperative Outcomes

All patients underwent primary curative resection by RATS or open surgery. Detailed information on the surgical approach and perioperative outcomes is given in Table 3. There were three planned conversions in the RATS group. Since R0 resection was achieved for all tumors in the RATS group, only R0 resections were included in the open surgery control. The median operation time was significantly shorter in the open surgery group (104 vs. 88 min, *p *= 0.0305). Extended resections involving the lung, pericardium, phrenic nerve, and innominate vein occurred more frequently in the open surgery group (22.9% vs. 51.7%, *p* = 0.0166). The median duration of a chest tube and the median length of stay were both significantly shorter in the RATS group (*p *< 0.0001 each). Postoperative complications, as outlined in Table 3, occurred more frequently in the open surgery group (cumulative incidence of 8.6% vs. 37.9%, *p* = 0.0047). Notably, significantly more patients in the open surgery group underwent adjuvant radiation therapy (2.9% vs. 55.2%, *p* < 0.0001).

**Table 3:** Surgical data and postoperative outcomes.

	RATS	Open surgery	*p* value
Incision			
Thoracotomy	—	22 (76%)	
Sternotomy	—	7 (24%)	
Side of approach			
Right	9 (26%)	7 (24%)	
Left	26 (74%)	15 (52%)	
Median	—	7 (24%)	
Planned conversion	3 (8.6%)	—	
Extended resections^a^, total	8 (22.9%)	15 (51.7%)	**0.0166**
Lung	4 (11.4%)	11 (37.9%)	
Pericardial	7 (20.0%)	10 (34.5%)	
Phrenic nerve	2 (5.7%)	5 (17.2%)	
Innominate vein	0	1 (3.4%)	
R0 resection	35 (100%)	29 (100%)^b^	
Operating time, min	104 (89−163)	88 (68−128)	**0.0305**
Console time, min	72 (55−96)	—	
Duration of chest tube, days	1 (1−2)	4 (3−5)	**< 0.0001**
Duration of postoperative stay, days	4 (3−6)	10 (8.5−12.5)	**< 0.0001**
Postoperative complications^a^, total	3 (8.6%)	11 (37.9%)	**0.0047**
Pneumonia	1 (2.9%)	2 (6.9%)	
Pleural effusion	0	2 (6.9%)	
Phrenic nerve palsy	2 (5.7%)	7 (24.1%)	
Recurrent laryngeal nerve palsy	0	2 (6.9%)	
Adjuvant radiotherapy	1 (2.9%)	16 (55.2%)	**< 0.0001**

*Note:* Surgical data and postoperative outcomes. Values are given as median (interquartile range) or *n* (%). *p* values show the result of the Chi‐square test for categorical variables and the Mann−Whitney test for continuous variables. Bold values indicate statistically significant.

^a^ Categories are not mutually exclusive.

^b^ Only patients following R0 resection were included in the open surgery control group according to selection criteria.

### 3.3 Long‐Term Survival and Recurrence

**Figure 2:**
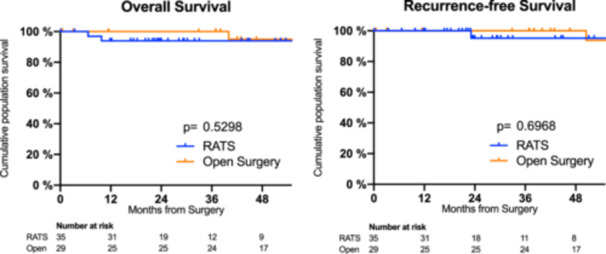
Kaplan−Meier plots of overall survival (OS) and recurrence‐free survival (RFS) comparing RATS and open surgery. *p* values are given for the log‐rank (Mantel−Cox) test.

The median follow‐up times were 28 months (interquartile range, IQR: 20−45 months) in the RATS group and 69 months (IQR: 37−95 months) in the open surgery control group. Figure 2 shows Kaplan−Meier plots of overall survival and disease‐free survival comparing the RATS and open surgery groups. We observed no differences in overall survival and recurrence‐free survival between the two surgical procedures. In all patients included in the study, we observed a 5‐year recurrence‐free survival of 96.9%. There were two patients with pleural disseminated tumor recurrence, one patient with a Type B1‐Thymoma in TNM Stage I who initially underwent RATS, and one patient with a Type AB‐Thymoma in TNM Stage I after thoracotomy.

## 4 Discussion

The use of RATS for the surgical treatment of large thymomas is controversial due to concerns about oncologic efficacy compared to open surgery. Few studies have been published on robotic‐assisted surgery for large thymomas > 50 mm in size. Jiang et al. reported that robotic‐assisted surgery for large mediastinal tumors is safe and effective compared with video‐assisted thoracoscopy and open surgery but pointed out that long‐term follow‐up to evaluate oncologic efficacy is lacking [15]. Similarly, Ye et al. pointed out the need to evaluate oncological outcomes, citing short follow‐up as a major limitation in their study of robotic versus transsternal thymoma resection [16]. In a recent study, Huang et al. investigated the resection of thymomas by RATS in a large, retrospective cohort and reported no increased risk of recurrence in patients with thymomas measuring > 50 mm, concluding that extended thymectomy is technically feasible, safe, and adequate for treating large resectable thymomas [17]. However, a comparison to open surgery, previously considered the gold standard for oncologic resection, was not included. Our data are consistent with recent reports on the perioperative outcomes of RATS—we also report fewer complications, a shorter duration of chest drainage, and a shorter length of stay compared to open surgery. Furthermore, we were now able to add insights into the risk of early recurrence associated with competing surgical approaches.

In this cohort, surgical treatment with RATS was not associated with an increased incidence of recurrence. Overall, the prognosis was excellent with a pooled 5‐year disease‐free survival of 96.9%, comparable to previous reports [18, 19]. In the comparative survival analysis, OS and DFS were similar in the RATS cohort and the open surgery cohort. The median follow‐up time of 28 months in the RATS group may be insufficient for a comprehensive view of the postoperative course of thymoma but is sufficient to assess early recurrence in relation to the surgical approach. Our data complement previous findings by Kang et al., who reported equivalent long‐term outcomes of RATS for smaller thymomas [18]. Similarly, previous studies have also examined oncologic outcomes after VATS thymectomy and found no increased incidence of recurrence associated with the minimally invasive technique [7, 20].

Some surgeons state a tumor size of 5 or 8 cm as the critical upper limit for minimally invasive VATS and RATS resections of thymic tumors [18, 20, 21]. In contrast to these reports, we do not consider tumor size as a main contraindication for the decision to perform RATS surgery, but rather a local extension and possible infiltration into the vena cava, pulmonary artery, trachea, esophagus, or atrium [14, 22]. While tumor infiltration into the lung and pericardium can be technically safely managed during RATS, invasion into the aorta, pulmonary artery, myocardium, trachea, or esophagus is considered a contraindication [18, 22].

Interestingly, extended resections were performed more frequently in the open surgery group, even though tumors were not further advanced locally. Although this is impossible to evaluate in the retrospective setup, we believe that the excellent visualization and fine dissection technique in RATS allows for more precise preparation of adhesions between the tumor and surrounding structures. In open surgery, this might simply have prompted the surgeon to perform extended resections, while RATS can offer the possibility to preserve surrounding structures that are only tightly adhesive but not infiltrated.

Postoperative radiotherapy (PORT) has been shown to influence survival of thymoma patients and must therefore be considered a potential confounder when assessing long‐term outcomes after surgical therapy [23, 24]. There is no prospective, multicenter evidence for PORT, and current practice varies widely [23]. Recent data suggest that PORT provides no survival benefit for Stage I thymoma, while its benefit for Stages II and III remains unclear [23, 24, 25]. Over the past decade, there has been a worldwide trend to reduce the use of PORT and reserve it for high‐risk cases [5, 23]. In this study, this trend is reflected in the significantly higher rate of PORT in the open surgery group from 2010 onwards, while RATS surgeries after 2018 are affected by the trend toward less frequent use of PORT. Our study is not designed to evaluate the benefit of radiation, but the survival data seem to indicate that the reduced use of PORT in the RATS group did not lead to an increased recurrence rate.

Although data was collected prospectively within a robotic surgery database, this study is limited by its retrospective design that possibly introduces selection bias in the indication for surgery. Especially differences in tumor size remain a possible confounder and selection bias, but because of the extreme rarity of the disease, the limited sample size significantly hindered attempts to mitigate those biases by performing propensity matching. However, the groups compared were similar in most relevant parameters. While the median follow‐up time of 28 months in the RATS group might be limited to display the postoperative course of thymoma comprehensively, it is by any means sufficient to evaluate early recurrence associated with the surgical approach. Since the introduction of RATS at our institution in 2018, increasingly larger thymomas have been operated by RATS as surgical expertise grew. This inevitably leads to the fact that most thymoma patients operated by open surgery date from the beginning of the study period, while those treated with RATS have been operated more recently. This time bias is also reflected in a shift regarding adjuvant radiotherapy over the last decade.

## 5 Conclusion

RATS thymectomy is a safe technique for the treatment of large thymomas with excellent oncologic outcomes comparable to those of open surgery. There is no evidence of early tumor recurrence associated with the use of RATS. The technical advantages, the favorable perioperative course as well as the low complication rate speak in favor for the use of RATS.

## Author Contributions

Martin E. Eichhorn, Hauke Winter, and Benedikt Niedermaier contributed to the study conception and design. Material preparation, data collection, and analysis were performed by Benedikt Niedermaier, Raffaella Griffo, and Thomas Muley. The first draft of the manuscript was written by Benedikt Niedermaier, and all authors commented on previous versions of the manuscript. All authors read and approved the final manuscript.

## Ethics Statement

The study was approved by the ethics committee of Heidelberg University under S174/2019.

## Conflicts of Interest

The authors report financial support by Intuitive Surgical for the research initiative “Robotic‐assisted thoracic surgery: impact on clinical and economic outcomes in a high volume European Thoracic Surgery Center” outside of the submitted work.

## Synopsis

Perioperative results and oncologic outcomes of robot‐assisted versus open surgery were investigated for large thymomas measuring > 5 cm. There is no evidence of early tumor recurrence associated with the use of RATS. The favorable perioperative course, including a shorter duration of chest drainage, shorter median length of stay, and the lower complication rate speak in favor for the use of RATS.

## Data Availability

The data underlying this article will be shared on reasonable request to the corresponding author.
